# TAP1 deficiency reshapes tumor antigenicity and enables CD8 T cell targeting in colorectal cancer

**DOI:** 10.1016/j.omton.2026.201294

**Published:** 2026-07-17

**Authors:** Audrey Merienne, Kathleen Ducoin, Cécile Deleine, Mathilde Deiber, Margot Machu, Edouard Leveque, Romain Oger, Emilie Duchalais, Pierre Fourquier, Céline Bossard, Nathalie Labarrière, Houssem Benlalam, Anne Jarry, Nadine Gervois-Segain

**Affiliations:** 1 Nantes Université, University Angers, CHU Nantes, INSERM, CNRS, Immunology and New Concepts in ImmunoTherapy, INCIT, UMR 1302/EMR6001, 44000 Nantes, France; 2 LabEx IGO and ImmuNE, Nantes Université, Nantes, France; 3 IRCM, University Montpellier, Inserm, ICM, CNRS, Montpellier, France; 4 University of Toulouse, Infinity, Inserm, CNRS, 31024 Toulouse, France; 5 Nantes Université, University Angers, INSERM, CNRS, Centre de Recherche en Cancérologie et Immunologie Intégrée Nantes-Angers, CRCI2NA, UMR 1307/EMR6001, 44000 Nantes, France; 6 Department of Digestive Surgery and IMAD, CHU Nantes, France; 7 Department of Digestive Surgery, Hôpital Privé Du Confluent, Nantes, France; 8 Department of Pathology, CHU Nantes, IHP Group, Nantes, France

**Keywords:** TAP1 deficiency, colorectal cancer, TILs, CD8 T cell response, 2D-3D co-culture models

## Abstract

Loss of components of the antigen processing machinery, such as the transporter associated with antigen processing 1 (TAP1), is a frequent immune escape mechanism in colorectal cancer (CRC). Although TAP1 deficiency impairs classical antigen presentation, it may also generate alternative T cell epitopes associated with impaired peptide processing (TEIPP) exploitable for immunotherapy. We investigated whether human CRC harbors functional CD8 T cells targeting TAP1-deficient tumor cells. TAP1 expression was analyzed by immunohistochemistry in tumors from 193 CRC patients (156 microsatellite-stable [MSS] and 37 microsatellite-instable [MSI]). CD8 tumor-infiltrating lymphocytes (TILs) were expanded from 61 CRC samples and tested for reactivity against a TAP1-deficient CRC cell line. 26% of CRC tumors displayed reduced or absent TAP1 expression, with no significant association with overall survival. CD8 TILs reactive to TAP1-deficient tumor cells were detected in 46% of patients and their frequency correlated with low TAP1 expression, particularly in MSI CRCs. A CD8 T cell clone selectively recognized and efficiently killed TAP1-deficient CRC cells in an HLA-B∗07:02-restricted manner, inducing robust tumor cell apoptosis in both 2D cultures and 3D spheroid models. These findings demonstrate that TAP1 deficiency reshapes tumor antigenicity and enables CD8 T cell targeting in CRC, supporting the development of immunotherapies directed against TAP1-deficient tumors.

## Graphical abstract

**Figure:**
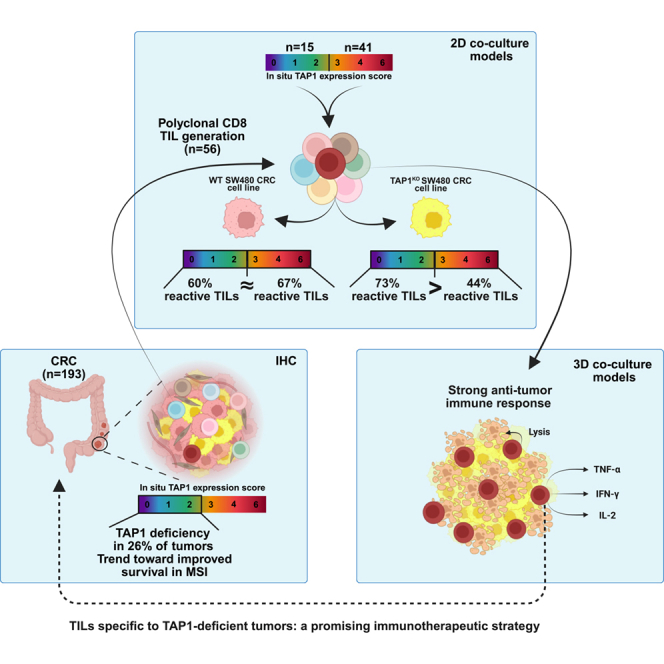

## Teaser

These results reveal that CD8 T cells infiltrating CRC tumors with low TAP1 expression frequently recognize TAP1-deficient tumor cells and develop strong antitumor potential monitored using a 3D model. This discovery opens up promising prospects for the development of tailored immunotherapies specifically designed to overcome immune escape in TAP1-deficient cancers.

## Introduction

Despite major advances in screening and treatment, colorectal cancer (CRC) remains one of the leading causes of cancer-related mortality worldwide, with a notable rise in its incidence rate in developing countries, reflecting changes in lifestyle and dietary habits.^1^ While immune checkpoint inhibitors (ICIs) have demonstrated remarkable efficacy in a subset of patients with microsatellite instable CRC (MSI, 15% of CRC), the majority of patients, particularly those with microsatellite stable tumors (MSS, 85% of CRC), derive little or no clinical benefit from current immunotherapies.^2^^,^^3^ Identifying novel immunotherapeutic targets capable of overcoming tumor immune resistance therefore remains a major unmet clinical need.

CD8^+^ cytotoxic T lymphocytes (CTLs) play a central role in tumor immune surveillance through recognition of tumor-derived peptides presented by HLA class I molecules. High densities of CD8^+^ T cells within tumors, captured clinically by the Immunoscore, correlate strongly with improved survival (supporting the better prognosis of MSI CRC compared to MSS) and outperform conventional tumor node metastasis (TNM) staging in prognostic accuracy. This underscores the therapeutic potential of harnessing T cell immunity in CRC.^4^^,^^5^

However, most CRC tumors display limited immunogenicity. MSI tumors harbor a high tumor-mutational burden and generate neoantigens capable of eliciting spontaneous T cell responses, whereas MSS tumors typically exhibit low mutational burden and poor antigenicity.^6^ In addition, tumor immune escape mechanisms, including defects in antigen presentation and the establishment of an immunosuppressive tumor microenvironment, further restrict the efficacy of immune checkpoint inhibitors (ICIs) and T cell-based immunotherapies.^7^^,^^8^^,^^9^ These constraints highlight the need to develop alternative immunotherapeutic strategies capable of generating effective T cell responses in MSS and MSI tumors, which could be combined with ICIs.

A major limitation of current immunotherapeutic strategies in CRC is the scarcity of truly tumor-specific antigens. Most shared tumor-associated antigens, such as the oncofetal antigen CEA (carcinoembryonic antigen) and cancer germline antigens, are also expressed in normal tissues and have shown limited clinical efficacy as vaccine targets, likely due to central tolerance.^10^^,^^11^^,^^12^^,^^13^ Tumor-specific antigens derived from mutations (neoantigens) are mainly found in MSI tumors with high tumor-mutational burden,^14^^,^^15^ whereas the majority of CRC patients display low immunogenicity.^16^ These limitations highlight the need for alternative approaches that do not rely exclusively on classical tumor-associated antigens or mutation-derived neoantigens.

Defects in the antigen processing machinery (APM), particularly involving the transporter associated with antigen processing (TAP), represent a frequent mechanism of immune evasion in CRC and other cancers.^17^ While TAP deficiency impairs conventional HLA class I antigen presentation, it also profoundly reshapes the tumor antigenic repertoire by enabling the presentation of unconventional peptides generated through alternative processing pathways.^18^ These tumor-restricted peptides, termed T cell epitopes associated with impaired peptide processing (TEIPP), are absent from healthy tissues and emerge specifically in TAP-deficient tumor cells, thereby constituting attractive targets for immunotherapy.

Importantly, in addition to HLA class I (HLA-I) and β2 microglobulin (β2m) expression defects, TAP1 downregulation has been reported in colorectal tumors and is associated with reduced immune recognition.^19^^,^^20^ Although TAP1 deficiency has long been considered a barrier to immunotherapy, recent studies suggest that TEIPP-based vaccination strategies may effectively target TAP-deficient tumors. In a recent clinical trial in patients with immune checkpoint-refractory non-small cell lung cancer (NSCLC), vaccination against a TEIPP derived from LRPAP1 (low-density lipoprotein receptor related protein-associated protein) induced specific CD8 T cell responses in the majority of patients and demonstrated preliminary evidence of clinical efficacy.^21^ These findings indicate that TAP-deficient tumors may represent a distinct and therapeutically exploitable tumor subset.

Despite these advances, the presence and frequency of naturally occurring CD8 T cells capable of recognizing and eliminating TAP1-deficient tumor cells in human CRC remain poorly characterized. Whether such T cells infiltrate primary tumors and retain functional antitumor activity has not been systematically investigated.

In this study, we aimed to determine the frequency of tumor-infiltrating CD8 αβ T cells reactive against a TAP1-deficient CRC cell line in a large cohort of CRC patients and to correlate this reactivity with TAP1 expression in autologous tumor tissues. Furthermore, we assessed the antitumor potential of TAP1-deficient tumor-specific CD8 T cells using both 2D cultures and 3D tumor spheroid models, providing a proof of concept for exploiting TAP1 deficiency as an immunotherapeutic vulnerability in CRC.

## Results

### TAP1 deficiency in CRC: A pattern observed in one-third of tumors that may influence the prognosis of MSI patients

**Figure 1:**
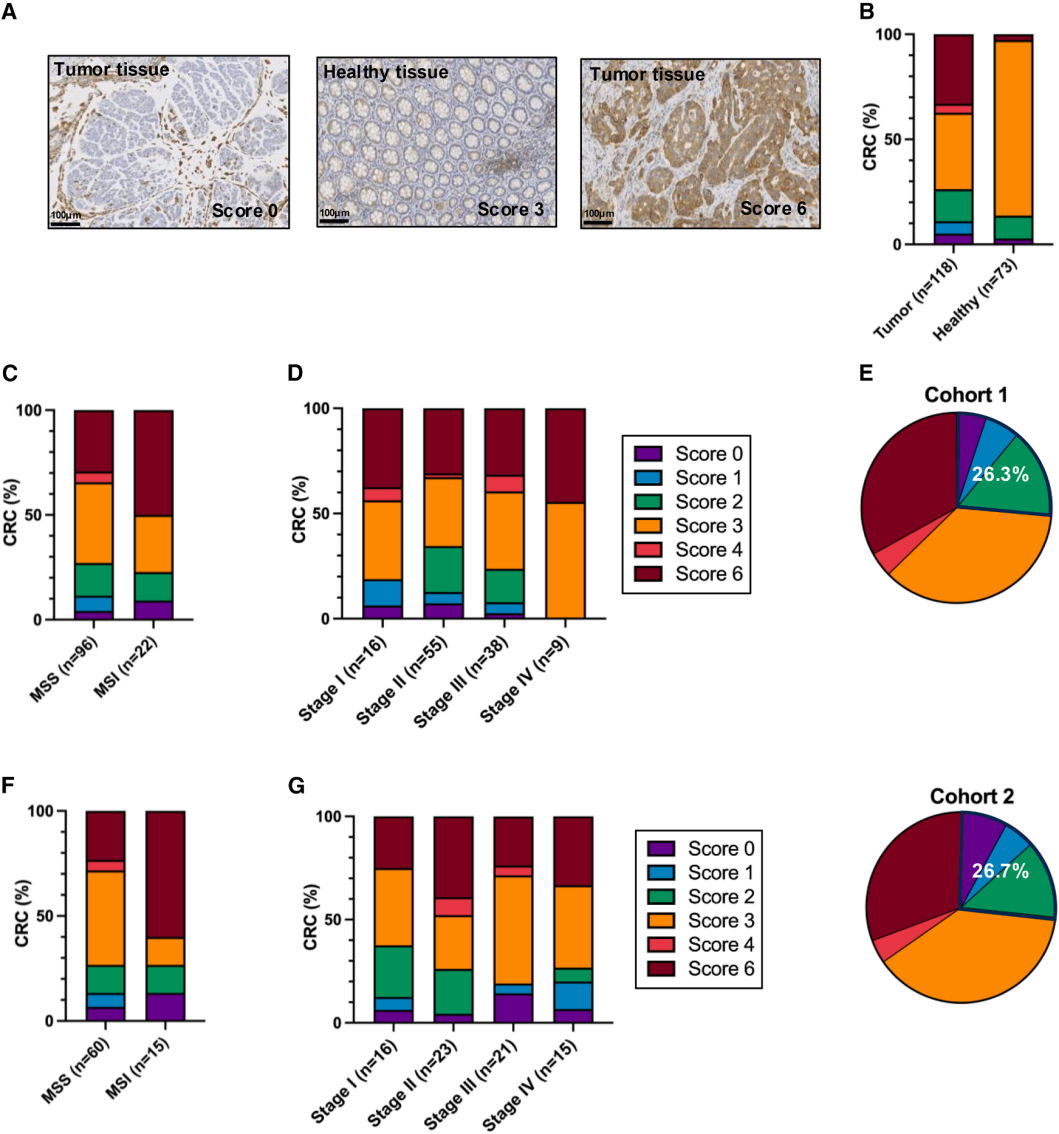
Relations between TAP1 expression and clinical parameters in colorectal cancer TAP1 IHC scores were determined by considering the percentage of positive tumor or normal epithelial cells and the staining intensity: 0 for no expression, 3 for moderate expression, and 6 for strong expression, as shown in Table S1. (A) Representative examples of the *in situ* TAP1 expression in tumor tissue (score = 0, left part of the image; score 6, right part of the image) and healthy tissue (score = 3, middle part of the image). (B) Stacked bar charts illustrating the distribution of patients with CRC (cohort 1) according to TAP1 expression levels in tumor (*n* = 118) and adjacent healthy tissues (*n* = 73). (C and D) Distribution among cohort 1 of TAP1 scores according to microsatellite status (C) and pTNM tumor stage (D). (E) Pie charts showing the distribution of TAP1 expression in tumors from cohort 1 and cohort 2 of CRC. (F and G) Distribution among cohort 2 of TAP1 scores according to microsatellite status (F) and pTNM tumor stage (G).

We first performed an *in situ* TAP1 expression in CRC by IHC (immunohistochemistry) on paraffin tumor sections from a first cohort of patients (*n* = 118 tumors, *n* = 73 paired adjacent healthy tissues). A semi-quantitative score (0–6) combined the percentage of stained tumor or normal epithelial cells and their staining intensity (Table S1). Representative images of IHC sections showing different levels of TAP1 expression are shown in Figure 1A. The overall analysis (Figure 1B) showed heterogeneous expression of TAP1 in tumors with strong expression (score 6, indicated in red) in 37% of cases and weak or no expression (score ≤2, outlined in red) in 26% of cases, of particular interest in this study. In comparison, in normal tissue adjacent to the tumor, TAP1 expression was very homogeneous and moderate in epithelial cells, with a predominant score of 3 (in 84% of cases). TAP1 levels were unrelated to MSS/MSI status (Figure 1C) or pTNM cancer stage (Figure 1D), although stage IV metastatic tumors strongly expressed TAP1 and never display TAP1 defect. No correlation was found with gender, age, location, or histological type (data not shown).

These findings were validated in a second cohort of 75 CRC patients, confirming a similar distribution of TAP1 expression (about 25% deficient tumors) (Figure 1E) and no significant association with MSS/MSI status (Figure 1F) or cancer stage (Figure 1G), though some tumors lacked TAP1 expression entirely.

**Figure 2:**
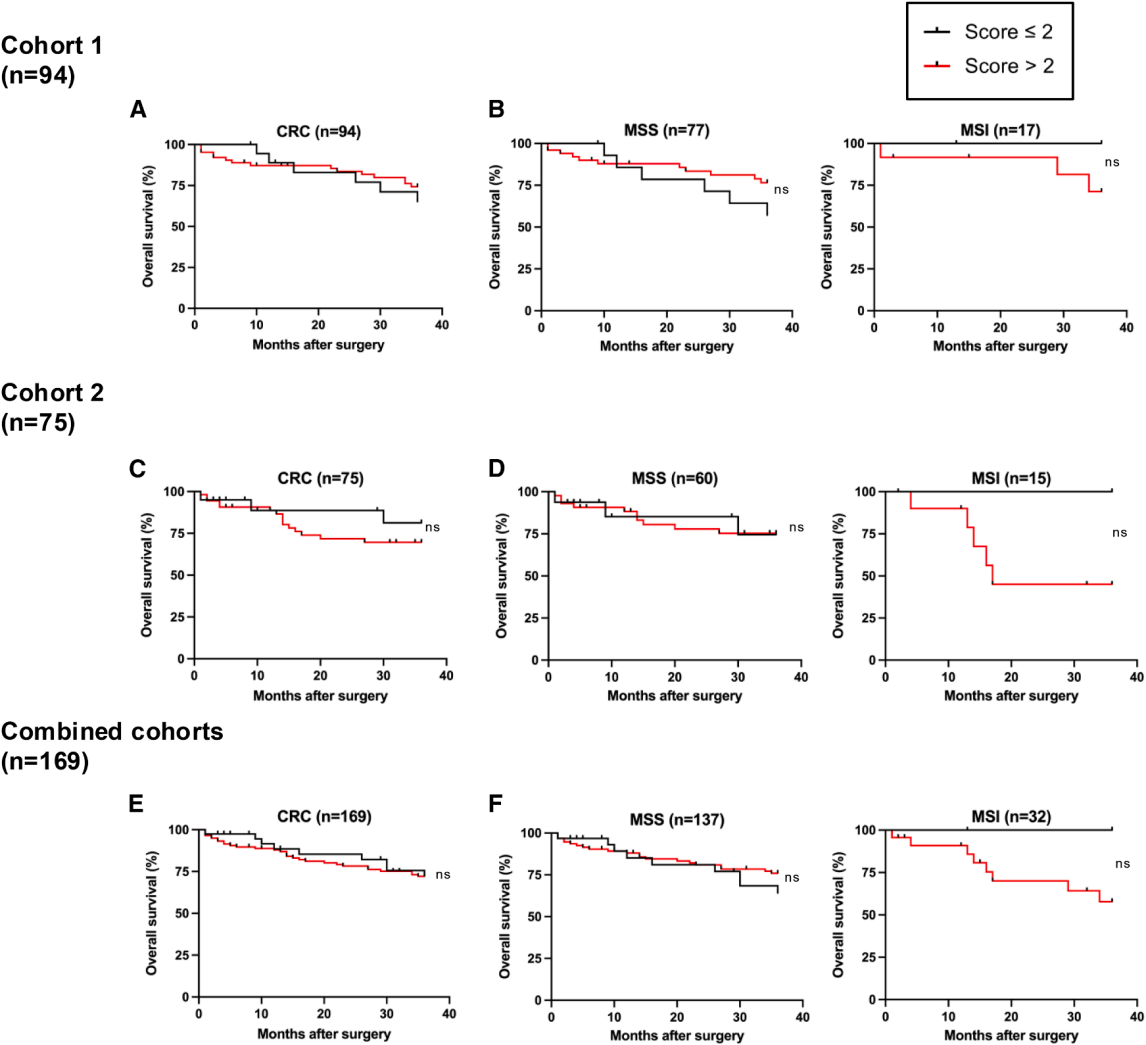
Correlation between TAP1 expression level and 3-year overall survival in CRC patients Kaplan-Meier survival curves compare overall survival at 3 years in patients whose tumors underexpress (score ≤2) or overexpress (score >2) TAP1 in cohort 1 (A), cohort 2 (C) or in both cohorts combined (E) and, (B, D, and F) according to MSS and MSI status (Mantel Cox log rank test).

Then, we examined whether there was a correlation between the level of TAP1 expression in the tumor and 3-year overall survival in CRC patients in cohort 1 (*n* = 94, Figures 2A and 2B) and cohort 2 (*n* = 75, Figures 2C and 2D) separately, and in the two cohorts combined (*n* = 169, Figures 2E and 2F). There was no overall statistical correlation (Mantel-Cox log rank test) between TAP1 expression and patient survival (Figures 2A–2C and 2E). However, MSI patients with low TAP1 expression tended to have better outcome in both cohorts analyzed separately (Figure 2B, cohort 1, *p* = 0.395; Figure 2D, cohort 2, *p* = 0.088) and even more pronounced when combined (Figure 2F *p* = 0.069). These results suggest that deficiency in TAP1 could be beneficial for MSI patients, by the generation of a new peptide repertoire, possibly recognized by CD8 TILs.

### Presence of CD8 TILs reactive to TAP1-deficient tumor cells

**Figure 3:**
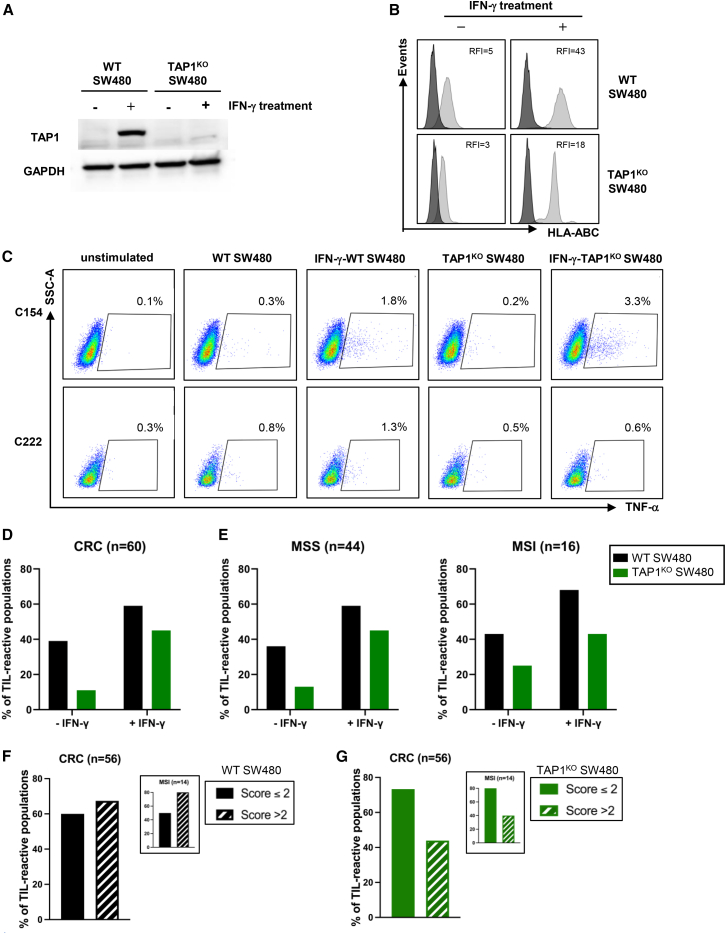
Existence of CD8 TILs reactive to TAP1-deficient CRC tumor cells. (A) WB analysis of TAP1 expression in WT and TAP1^KO^ SW480 cell lines, with or without 48-h pre-treatment with IFN-γ. GAPDH was used as a loading control. (B) Representative histograms showing HLA-ABC expression (gray) by WT and TAP1^KO^ SW480 cell lines, with or without 48-h pre-treatment with IFN-γ. Expression levels were measured by flow cytometry and expressed as relative fluorescence intensity (RFI). Controls are overlaid in black. (C–E) Representative dot plots illustrating TNF-α production by CD8 TILs from two patients (C154 and C222) after co-culture with WT or TAP1^KO^ SW480 cells, pre-treated or not for 48 h with IFN-γ. TNF-α production was assessed by flow cytometry and expressed as the percentage of TNF-α-positive CD8^+^ cells. Histograms comparing the frequency CD8 TILs reactive against WT and TAP1^KO^ SW480 pre-treated or not with INF-γ in the entire cohort (D) or according to the MSS/MSI status (E). (F and G) Frequency of WT (F) and TAP1^KO^ (G) SW480 cells (pre-treated with INF-γ)-reactive CD8 TILs stratified by TAP1 level *in situ* expression of tumors from which TILs were isolated (≤ score 2 and > score 3). The small insert includes a focus on MSI tumors.

To identify CD8 αβ TILs capable of responding to TAP1-deficient tumor cells and given the impossibility of generating autologous tumor cell lines, we chose to disrupt the *TAP1* gene, using CRISPR-Cas9 approach, in the SW480 CRC line presented the advantage to express common HLA-I alleles (HLA-A∗02:01, HLA-A∗24:02, HLA-B∗07:02, HLA-B∗15:18, HLA-Cw∗07:02, and HLA-Cw∗07:04). We thus generated a TAP1^KO^ (Knockout) SW480 cell line, validated by the absence of detectable TAP1 protein expression (WB) even after 48 h of treatment with IFN-γ (interferon-γ) (500 UI/mL) compared to the WT (wild type) cell line, in which, TAP1 is only expressed after IFN-γ-treatment, as already described in CRC lines^22^ (Figure 3A). As expected, TAP1^KO^ cells displayed defective basal HLA-I expression, which can nevertheless be restored after IFN-γ treatment, a prerequisite for the presentation of HLA/peptide complexes to CD8 αβ T lymphocytes (Figure 3B).

To assess anti-tumor T cell responses, TIL cultures were established from tumor samples of 61 CRC patients (45 MSS and 16 MSI). After *ex vivo* expansion, CD8 T cells were purified by magnetic sorting and subsequently used in functional assays evaluating the recognition of TAP1-deficient tumor cells.

Their reactivity against WT or TAP1^KO^ SW480 cell lines, preincubated or not for 48 h with IFN-γ, was assessed by intracellular TNF-α (tumor necrosis factor-α) production (representative examples in Figure 3C). The average frequency of TNF-α-producing CD8 T cells within polyclonal TIL populations was 0.54% ± 0.52% (range: 0%–4.25%) in response to TAP1^KO^ SW480 cells and 0.77% ± 0.70% (range: 0%–5.7%) in response to WT SW480 cells. To classify TIL populations as reactive or non-reactive, we used the median frequency of TNF-α-producing CD8 T cells observed across the cohort (0.3%) as a cutoff value. TIL populations exhibiting frequencies ≥0.3% were considered reactive. Overall, the results showed that 39% of CD8 TIL populations contain T cells reacting to the WT SW480 cell line, compared with 11.5% reacting to the TAP1^KO^ cell line, regardless of the microsatellite status of the corresponding tumor (Figures 3D and 3E). However, if the target is first pre-stimulated by IFN-γ, this difference narrows, with the percentages increasing to 59% and 46%, respectively (Figure 3D).

Among the 56 evaluable cases for which we had TAP1 expression scores in the primary tumor (IHC), we examined whether the presence of TILs reactive against the TAP1^KO^ SW480 cell line depended on the level of TAP1 expression in the corresponding tumor. Interestingly, we showed a correlation between the frequency of TIL populations reactive against TAP1^KO^ SW480 and low *in situ* expression of TAP1 in the corresponding tumor tissue (Table 1; Figure 3F).

**Table 1:** Correlation between the frequency of CD8 TILs reactive to TAP1^KO^ SW480 and *in situ* TAP1 expression score in the original tumor

	Reactivity of CD8 TIL populations	TAP1 score (*in situ*) of tumors from which TILs originate		Chi-square test
		Score ≤2	Score >2	
**CRC (*n* = 56)**	Yes	11	18	ns *p* = 0.051
	No	4	23	
**MSS (*n* = 42)**	yes	7	14	ns *p* = 0.292
	No	4	17	
**MSI (*n* = 14)**	yes	4	4	**∗*p* = 0,0404**
	No	0	6	

Indeed, for a low TAP1 score (≤2) in the tumor, 11 of the 15 TIL populations (73.3%) were reactive to the TAP1^KO^ cell line, compared with 18 of 41 (43.9%) for a higher score (greater than 2) (chi-square test, *p* = 0.051). This correlation was mainly attributed to MSI tumors (chi-square test, *p* = 0.040), although a similar trend was observed for MSS tumors (chi-square test, *p* = 0.292). Reinforcing the value of these findings, our data showed no difference in the frequency of WT SW480-reactive T cells between the two TIL groups defined according to TAP1 expression in the original tumors (60% vs. 67%), although there was a higher frequency of reactive T cells in MSI tumors with a high TAP1 score (80%) (Table S2; Figure 3G).

### Antitumor efficacy of a CD8 TIL T cell clone specific to the TAP1^KO^ SW480 cell line

**Figure 4:**
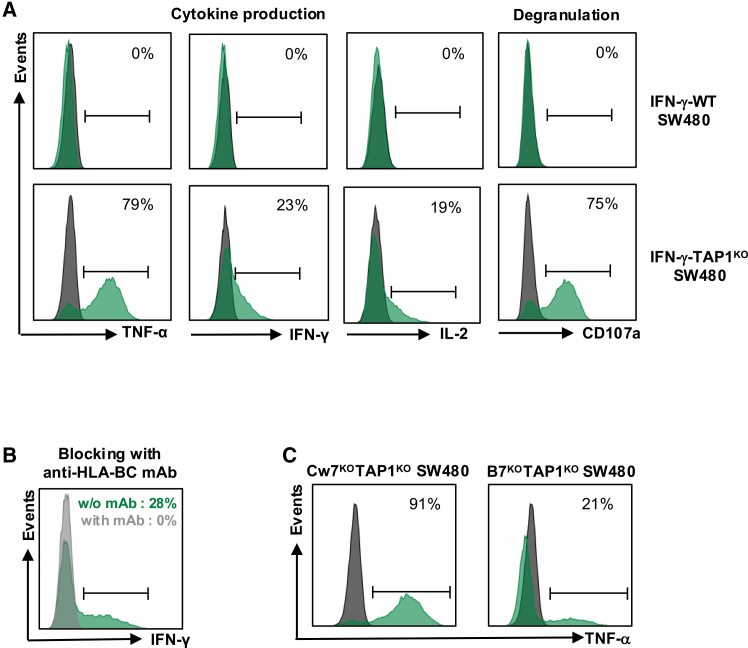
Functional characterization of a CD8 TIL clone specific for the TAP1^KO^ SW480 cell line Responses were assessed by flow cytometry and are expressed as the percentage of positive cells (green), with controls overlaid in black. (A) Representative histograms showing cytokine production (IFN-γ, TNF-α, and IL-2) and degranulation marker (CD107a) by the 91AE T cell clone after stimulation with WT or TAP1^KO^ SW480 cells pre-treated for 48 h with IFN-γ. (B) Representative histogram showing IFN-γ production by the 91AE T cell clone against IFN-γ pretreated TAP1^KO^ SW480 cells, either in the presence (gray) or absence (green) of the blocking anti-HLA-BC mAb (B.1.23.2). (C) Histogram showing the TNF-α production of the 91AE T cell clone following stimulation with HLA-B7^KO^TAP1^KO^ or HLA-Cw7^KO^TAP1^KO^ SW480 cell lines pre-treated with IFN-γ for 48 h.

In order to formally demonstrate the antitumor potential of CD8 T cells against TAP1-deficient tumor cells, we generated a T cell clone (named 91AE) from CD8 TILs of the TAP1-negative C91 tumor (IHC score 0, Figure 1A). The results of stimulation of this clone by WT or TAP1^KO^ SW480 cell lines (pretreated with IFN-γ, 48 h, 500 IU/mL) clearly showed exclusive recognition of the TAP1^KO^ cell line, as evidenced by its cytokine response (higher number of cells producing TNF-α, IFN-γ, and IL-2 and its degranulation capacity (high levels of cells expressing CD107a on their surface), as assessed by flow cytometry (Figure 4A). To determine the HLA-I allele restricting antigenic peptide recognition by this clone, we performed co-culture experiments in the presence of anti-HLA-I, -HLA-A2, -HLA-24 and -HLA-BC blocking mAbs. As shown in Figure 4B, anti-HLA-BC abolished the IFN-γ response of the 91AE T cell clone. Since C91 tumor and the SW480 cell line shared the HLA-B∗07:02 and HLA-Cw∗07:02 alleles, we performed a knockdown of either of these two alleles in the TAP1^KO^ SW480 cell line. The reactivity of the 91AE T cell clone toward these HLA^KO^ cell lines clearly showed a strong decrease in the T cell clone reactivity toward the TAP1^KO^ SW480 cell line deficient for HLA-B∗07:02 expression (from 79% to 21% of TNF-α-producing cells), thus defining the autologous HLA-B∗07:02 as the restricting HLA allele and excluding any allogeneic reactivity (Figure 4C).

**Figure 5:**
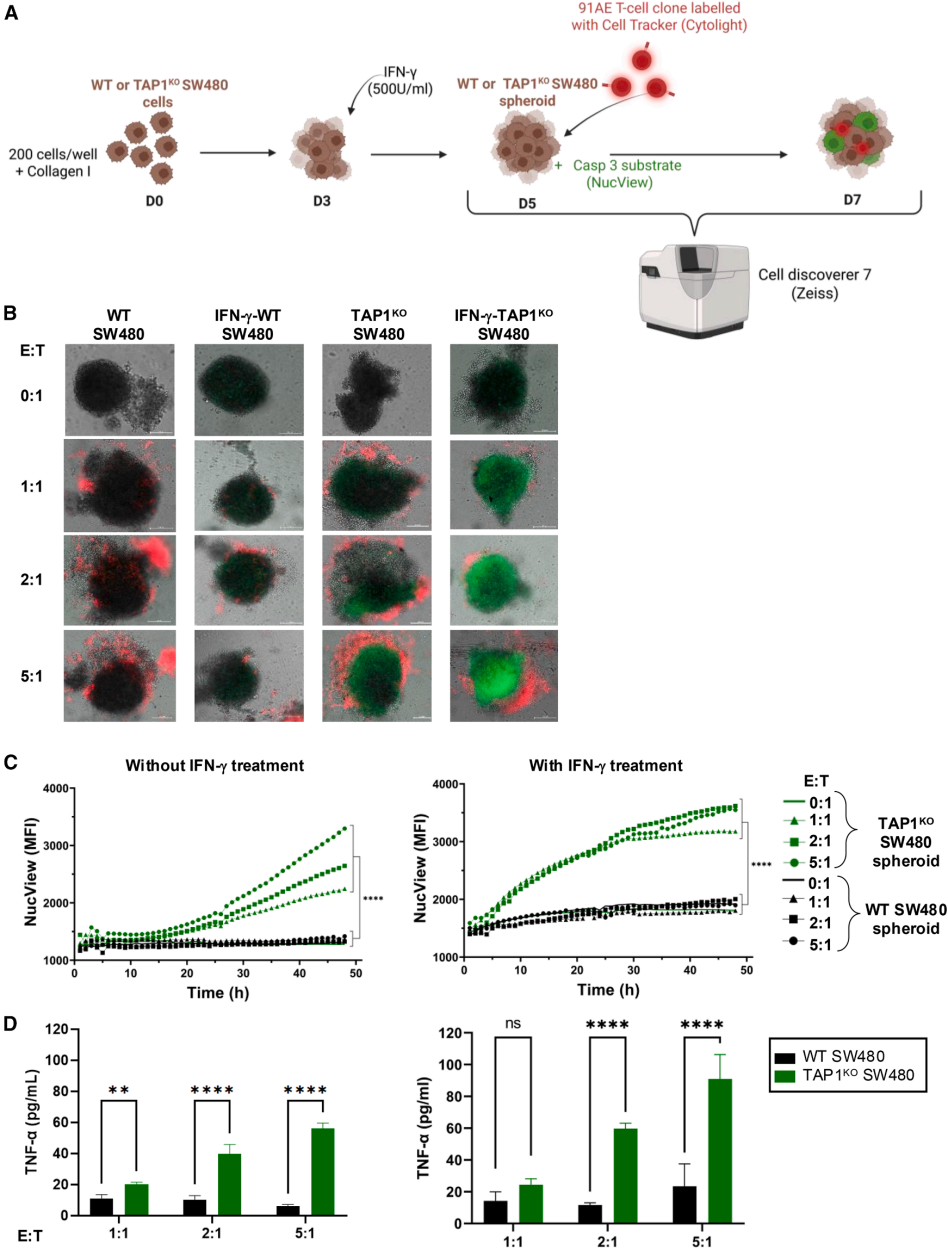
Anti-tumor activity of a 91AE T cell clone in a 3D spheroid model of TAP1-deficient CRC cells (A) Schematic representation illustrating the protocol used for generating 3D spheroids and monitoring T cell infiltration and tumor cell apoptosis. (B) Representative images of spheroids acquired using the automated high-resolution Cell Discoverer 7 microscope showing WT or TAP1^KO^ SW480 spheroids, pre-treated or not with IFN-γ, after 48 h co-culture with the 91AE T cell clone at various E:T ratios (0:1, 1:1, 2:1, 5:1). Lymphocytes are detected in red (Cytolight) and tumor cell apoptosis in green (Nucview caspase 3). (C) Tumor cells apoptosis within WT (black) or TAP1^KO^ (green) SW480 spheroids pre-treated or not with IFN-γ was assessed by calculating the mean fluorescence intensity (MFI) of NucView using Arivis software, measured every hour for 48 h, in the presence or absence of the 91AE T cell clone (two-way ANOVA, ∗∗∗∗*p* < 0.0001). (D) TNF-α secretion in the supernatant of co-cultures of WT (black) or TAP1^KO^ (green) SW480 spheroids (pre-treated or not with IFN-γ) with or without the 91AE T cell clone was quantified by ELISA after 48 h (two-way ANOVA, ∗∗*p* < 0.003, ∗∗∗∗*p* < 0.0001).

We then developed a 3D spheroid model from WT and TAP1^KO^ SW480 cell lines with time-lapse imaging of living cells (Cell Discover 7, Zeiss) (protocol described in Figure 5A). On the fifth day, 2 days after adding IFN-γ, some spheroids were dissociated to assess HLA-I expression and the number of cells per spheroid (which turned out to be between 15,000 and 20,000) in order to adjust the number of cell tracker-labeled 91AE T cells for different effector:target (E:T) ratios (1:1, 2:1, and 5:1). A caspase-3 substrate was also added to the wells to monitor tumor cell apoptosis. The WT or TAP1^KO^ SW480 spheroids (with or without IFN-γ supplementation) and were co-cultured in presence of labeled T cells for 48 h in a videomicroscope (CD7), and images were recorded every hour and analyzed.

We first showed that IFN-γ treatment effectively increased the expression of HLA-I molecules on the surface of all tumor cells in the spheroids, and notably in the TAP1^KO^ SW480 model (Figure S1). The time-lapse images of WT and TAP1^KO^ SW480 spheroids, pretreated or not with IFN-γ from day 3 to day 5, with or without the addition of clone T 91AE on day 5 (at different E:T ratios), are shown in Figure 5B, at the end of the experiment on day 7. All time-lapse images obtained from the spheroids at various time points after adding (or not) of the T cell clone are presented in supplemental figures (Figures S2A and S2B, with or without IFN-γ, respectively). Movies showing the evolution, from D5 to D7, of WT and TAP1^KO^ SW480 spheroids (treated with IFN-γ on D3) and in the presence of the T cell clone (added at an E:T ratio of 5:1 on D5) are also provided (Videos S1 and S2, respectively). We observed, that without the T cell clone, WT and TAP1^KO^ SW480 spheroids retained their round and dense shape, whereas upon co-culture with the specific T cell clone, only TAP1^KO^ spheroids lost their integrity, particularly in the presence of IFN-γ. Furthermore, while the results revealed relatively similar infiltration of the T cell clone (in red) in both spheroid models, tumor cell apoptosis (in green) was induced only in TAP1^KO^ SW480 spheroids, in a dose-dependent manner relative to the amount of clone added. Indeed, monitoring tumor cell labeling by caspase-3 activation revealed an increase in Nucview MFI over time in TAP1^KO^ SW480 spheroids, even greater under IFN-γ-treatment, reflecting apoptosis of these cells (Figure 5C). Finally, on day 7, we collected the supernatants from the different co-culture conditions to assess the presence of TNF-α by ELISA (enzyme-linked immunosorbent assay). As shown in Figure 5D, TNF-α secretion was predominantly detected in the supernatants of IFN-γ-treated TAP1^KO^ SW480 spheroids in the presence of clone 91AE, and increased proportionally with the number of T cells added. Once again, TNF-α production, although lower, was also detectable in the supernatants of TAP1^KO^ SW480 spheroids without IFN-γ. Overall, these results demonstrate the anti-tumor potential of a T cell clone derived from TILs in a 3D spheroid model of a TAP1-deficient CRC cell line.

Document S1. Supplemental materials and methods, Figures S1 and S2 and Tables S1 and S2

Video S1. Time-lapse live-cell imaging (CD7) of IFN-γ-treated WT SW480 tumor spheroids co-cultured with cytolight-labeled 91AE T cell clone, showing T-cell infiltration and NucView caspase-3-positive apoptotic tumor cells over 48h

## Discussion

Tumor cells frequently modulate TAP1 expression, particularly under immune selective pressure, to promote immune evasion and tumor progression.^23^ TAP1 deficiency is therefore widely regarded as a classical mechanism of escape from CD8 T cell-mediated immunity by impairing antigen presentation through the conventional HLA class I pathway. Paradoxically, impairment of this pathway can give rise to alternative antigen processing routes that generate unconventional peptides, termed TEIPPs, derived from normal proteins and selectively presented on TAP-deficient tumor cells.^24^

The main objective of this study was to determine whether TAP1-deficient CRC tumors harbor tumor-infiltrating CD8 T cells capable of recognizing and eliminating TAP1-deficient cancer cells, to assess the relationship between this reactivity and TAP1 expression in tumor tissues, and to evaluate the functional antitumor potential of such T cells.

Consistent with previous reports in CRC and other cancers, we observed marked heterogeneity of TAP1 expression in tumor tissues compared with adjacent normal mucosa.^19^^,^^25^^,^^26^^,^^27^ While approximately 35% of CRC tumors exhibited strong TAP1 expression, nearly one-quarter displayed low or absent TAP1 levels, defining a distinct tumor subset potentially amenable to alternative immunotherapeutic strategies. These findings were confirmed in two independent patient cohorts and are in agreement with observations made in melanoma, prostate cancer, breast cancer, glioblastoma, and NSCLC.^28^^,^^29^^,^^30^^,^^31^^,^^32^^,^^33^

In line with Ling et al.,^26^ we found no significant difference in TAP1 expression between MSS and MSI tumors, although fewer TAP1 defects were observed in stage IV cancers (cohort 1). TAP1 expression was not significantly associated with 3-year overall survival in the combined cohorts, regardless of microsatellite status. These results are consistent with those reported by Kasajima et al.^25^ but differ from Ling et al.,^26^ who described an association between TAP1 downregulation and poor prognosis. Such discrepancies may be explained by differences in TAP1 scoring thresholds and by the absence of MSS/MSI stratification in their analysis. Interestingly, we observed opposite survival trends according to microsatellite status, with a tendency toward improved survival in MSI tumors and poorer outcome in MSS tumors with low TAP1 expression. Similar context-dependent associations between TAP1 expression and survival have been reported in other cancers.^20^^,^^33^

Using a CRISPR-Cas9-generated TAP1^KO^ SW480 CRC cell line, we demonstrated for the first time that a substantial proportion of CRC-derived CD8 TIL populations are capable of recognizing TAP1-deficient tumor cells. Approximately half of the TIL populations tested showed reactivity against TAP1^KO^ cells, independently of microsatellite status. Importantly, the frequency of TAP1^KO^-reactive TILs correlated with TAP1 expression levels in the corresponding primary tumors, with 73% of TILs from TAP1-low tumors displaying reactivity compared with 44% from TAP1-high tumors. This correlation was mainly driven by MSI tumors.

Conversely, TIL reactivity against the wild-type SW480 cell line was similar or higher in TAP1-proficient tumors, particularly in MSI cases, indicating that TAP1 deficiency selectively shapes the tumor-reactive T cell repertoire rather than globally increasing immunogenicity. The significant association between TAP1 deficiency and the presence of TAP1^KO^-reactive TILs in MSI tumors, not previously reported, may provide a mechanistic explanation for the trend toward improved survival observed in this subgroup. We hypothesize that the high mutational burden characteristic of MSI tumors may favor upregulation of components involved in alternative antigen processing pathways, thereby promoting TEIPP presentation. Supporting this hypothesis, transcriptomic analyses from public CRC datasets reveal increased expression of metalloproteases and signal peptide peptidases involved in alternative processing pathways in MSI tumors. Further investigation of these mechanisms may clarify the link between TAP1 deficiency and enhanced immune recognition in MSI CRC.

To further validate the functional relevance of TAP1-deficient tumor-reactive T cells, we generated a CD8 T cell clone from a TAP1-deficient CRC tumor. This clone displayed strong and selective activation upon stimulation with TAP1^KO^ tumor cells, as evidenced by cytokine production (TNF-α, IFN-γ, and IL-2) and degranulation, and showed no reactivity toward the TAP1-proficient counterpart. HLA restriction analysis confirmed that this response was mediated through the shared HLA-B∗07:02 allele, excluding an allogeneic reaction.

The antitumor activity of this clone was further confirmed using a 3D tumor spheroid model, in which complete apoptosis of TAP1^KO^ tumor cells was observed. Notably, tumor cell apoptosis was also detected in the absence of exogenous IFN-γ, albeit at lower levels. This suggests that initial low-level recognition of TAP1-deficient tumor cells may induce IFN-γ secretion by the T cells, leading to upregulation of HLA-I/peptide complexes and amplification of tumor cell killing. These findings highlight the potential role of the tumor microenvironment in enhancing the efficacy of TAP1-deficient tumor-reactive T cells.

The presence of significant frequencies of CD8 TILs recognizing TAP1-deficient tumor cells underscores the importance of identifying the underlying TEIPP antigens. Initial TEIPPs were described in murine models, such as epitopes derived from Trh4.^34^ In humans, TEIPP antigens have been predominantly described in HLA-A restricted contexts. The first example was reported by Fathia Mami-Chouaib’s team, who identified a TEIPP derived from the signal sequence of preprocalcitonin and recognized by an HLA-A02:01-restricted CD8 T cell clone isolated from lung carcinoma TILs.^35^^,^^36^ This was followed by the identification of multiple HLA-A02:01-restricted TEIPPs by Thorbald van Hall’s team using *in silico* prediction approaches, some of which were able to stimulate diverse CD8 T cell repertoires from PBMC, including an LRPAP1-derived epitope that progressed to a first therapeutic vaccination trial.^21^^,^^24^^,^^37^ Most TEIPPs characterized to date are restricted to HLA-A alleles, whereas our identification of responses in an HLA-B context broadens the potential applicability of this antigen class for immunotherapy.

Although the precise processing pathways of TEIPPs are not fully elucidated, several alternative routes involving the proteasome, signal peptidases, signal peptide peptidase, metalloproteases, furin, and endosomal cathepsins have been proposed.^18^^,^^38^^,^^39^^,^^40^ Importantly, TEIPPs possess key features that make them attractive therapeutic targets: they are absent from normal tissues, are selectively presented by TAP-deficient tumor cells, and escape central tolerance, allowing the generation of functional T cell repertoires. Their shared nature further supports their potential use in broadly applicable immunotherapeutic strategies, given the frequent TAP downregulation in various tumors.^17^^,^^18^^,^^40^

A recent phase 1/2 clinical trial in patients with immune checkpoint-refractory non-small cell lung cancer evaluated vaccination with long LRPAP1-derived peptides and demonstrated good tolerability, induction of TEIPP-specific CD8 T cells in 83% of patients, and objective clinical responses in a subset of cases.^21^^,^^37^ These results provide clinical support for the feasibility of targeting TEIPPs in TAP-deficient tumors.

The identification of functional CD8 TILs capable of selectively recognizing TAP1-deficient tumor cells has important therapeutic implications. Our findings indicate that TAP1 loss, classically viewed as a mechanism of immune escape, also creates an actionable vulnerability that can be exploited by CD8 T cell-based immunotherapies. The strong antitumor activity of a TAP1-deficient tumor-specific CD8 T cell clone in both 2D and 3D models supports the feasibility of targeting this tumor subset using adoptive T cell transfer or TEIPP-based vaccination strategies.

While the precise TEIPP recognized by the 91AE clone remains to be identified, the present study establishes a robust proof of concept that TAP1-deficient CRCs are not immunologically silent but can be efficiently targeted by tumor-reactive CD8 T cells. Importantly, this strategy may be particularly relevant for tumors resistant to immune checkpoint blockade, including a fraction of microsatellite-stable CRCs. Together, these data provide a strong rationale for the development of immunotherapeutic approaches specifically tailored to TAP1-deficient tumors.

## Methods

### Patients

A total of 193 patients with CRC operated without prior chemo- or radio-therapy at the Centre Hospitalier Universitaire de Nantes or Hôpital privé du Confluent (France) between 2015 and 2024 were included. Pathological staging followed the 8th edition of the UICC pTNM classification for CRC, and histological subtyping was reviewed according to the 4th edition of the WHO Classification of Tumors of the Digestive System.

All tissue samples were handled in accordance with French Ethics Committee for Human Tissue Research. Our biocollection is registered with the French Ministry of Higher Education and Research (DC-2014-2206) and approved by the Ethics Committee (CPP Ouest IV-Nantes). This study complied with the Declaration of Helsinki, and all patients provided written informed consent.

Clinicopathological features of patients from the two cohorts (cohort 1, *n* = 118; cohort 2, *n* = 75) are summarized in Table 2.

**Table 2:** Clinicopathological characteristics of CRC patients

	Cohort 1 *n* = 118	Cohort 2 *n* = 75
**Sex, n (%)**		

Female	51 (43.2%)	27 (36%)
Male	67 (56.8%)	48 (64%)

**Age at inclusion, mean (range)**		

–	69 (28–95)	70 (35–84)

**Microsatellite status, n (%)**		

MSS	96 (81.4%)	60 (80%)
MSI	22 (18.6%)	15 (20%)

**Tumor location, n**		

Right colon	52	31
Transverse colon	0	3
Left colon	51	32
Rectum	15	9

**pTNM stage (IUCC), n**		

Stage I	16	16
Stage II	55	23
Stage III	38	21
Stage IV	9	15

**Histological subtypes, n**		

Adenocarcinoma NOS	99	69
Mucinous adenocarcinoma	19	6

Abbreviations: pTNM: Pathological Tumor Node Metastasis; UICC: Union for International Cancer Control

### Microsatellite instability and DNA mismatch repair status

Mismatch repair or microsatellite instability status was determined for diagnostic purposes by immunohistochemistry and, if necessary, by PCR, as described previously.^41^

### IHC and immunostaining scores

Immunostainings were performed on 3 μm formalin-fixed paraffin-embedded sections from a representative tumor block (on whole sections for cohort 1 and on TMA (tissue microarray) for cohort 2) to assess TAP1 expression by tumor and paired-normal cells, using the following primary antibody (Ab) anti-human TAP-1 (CliniSciences, polyclonal rabbit, RRID:AB_373231).

Immunostainings were performed on an Autostainer Link 48 (Agilent Technologies) using standard protocols with antigen retrieval. Detection used peroxidase/diaminobenzidine Envision system (Agilent Technologies) with hematoxylin counterstain. After scanning the slides (NanoZoomer, Hamamatsu Photonics, Massy, France), TAP1 was semi-quantitatively scored (0–6 scale) in tumor and normal epithelial cells based on % positive cells and intensity (Table S1).

### Tumor cell lines and culture

The WT SW480 cell line (RRID: CVCL_0546) was established from a primary Dukes’ stage II MSS colon adenocarcinoma. This WT and other derived and KO generated cell lines were cultured in RPMI 1640 medium (Gibco) with 10% (FBS (Eurobio), 2 mM GlutaMAX-I (Gibco), 100 U/mL penicillin (Gibco) and 0.1 mg/mL streptomycin (Gibco).

### Generation and validation of KO SW480 cell lines

The TAP1^KO^ SW480 CRC cell line was generated using CRISPR-Cas9 technology with a single-guide RNA (sgRNA) targeting exon 1 of *TAP1*, designed and cloned into a lentiCRISPR vector by T. van Hall’s team.^24^ In brief, the WT SW480 cell line was transduced with a lentivirus (kindly provided by T. van Hall) for 24 h, selected with puromycin and sorted by by flow cytometry (BD FACSAria III cell sorter) as HLA-ABC^low^ cells using anti-HLA-ABC-FITC (BD Biosciences, clone G46–2.6, RRID: AB_395935) monoclonal Ab (mAb). The loss of HLA-I expression on sorted cells was verified by flow cytometry, and the absence of TAP1 was confirmed by WB using standard protocols.

The generation procedure of HLA-B∗07:02^KO^ and HLA-Cw07:02^KO^ SW480 cell lines and its validation are described in the supplemental materials and methods.

### Generation of CD8 TILs and clones

#### Generation of TILs

TILs from 61 CRC patients, 45 MSS and 16 MSI, were obtained after culture of tumor fragments (approximately 1 mm^3^) during 3 weeks in RPMI 1640 medium supplemented with 8% human serum (Dutscher), 2 mM GlutaMAX-I (Gibco), 100 U/mL penicillin, 0.1 mg/mL streptomycin, 1 μg/mL Amphotericin B (Sigma-Aldrich), 0.1 mg/mL gentamycin (Sigma-Aldrich) and 150 U/mL of human rIL-2 (Proleukin, Novartis), as previously reported.^42^ Fragments were removed at the end of the 3 weeks by filtration through a 40 μm cell filter.

#### Sorting of CD8 TIL subpopulations

CD8^+^ TILs were collected by magnetic cell sorting using the REAlease CD8 MicroBead kit by positive selection (Miltenyi Biotec) according to the manufacturer’s instructions. After cell sorting, CD8^+^ TILs were amplified by stimulation with phytohemagglutinin-L (Sigma Aldrich) and 150 U/mL human rIL-2, in the presence of irradiated allogeneic feeder cells (PBMCs and B-EBV cells), as described previously.^43^ The purity of each subpopulation was checked before and after cell sorting and regularly by flow cytometry.

#### T cell clone generation

The T cell clone (91AE) was obtained by limiting dilution cloning of patient C91’s polyclonal population of CD8 TILs and amplified as described above. This T cell clone was selected on the basis of its ability to specifically recognize the TAP1^KO^ SW480 cell line.

### Reactivity assay of TILs against CRC cell lines

SW480 CRC cell lines (WT and TAP1^KO^) were pretreated or not with 500 U/mL of recombinant human IFN-γ (Eurobio) for 48 h prior to a 5-h co-culture with polyclonal CD8 TILs and/or the 91AE T cell clone T cell clone (E:T 1:2) in the presence of 10 μg/mL brefeldin A. Cells were then stained with anti-CD8-PE (BioLegend, RPA-T8, RRID:AB_314126) in PBS/0.1% BSA (30 min), fixed in 4% paraformaldehyde (Electron Microscopy Sciences 15714-S), permeabilized in PBS/0.1% BSA/0.1% saponin (Sigma-Aldrich), and stained 30 min with anti-TNF-α-APC (clone Mab11, RRID:AB_315264), anti-IFN-γ-APC (clone B27, RRID:AB_315443), or anti-IL-2-APC (clone MQ1-17H12, RRID:AB_315098) (BioLegend). For CD107a degranulation, cells were stimulated 3 h without brefeldin A with anti-CD107a-AF647 (BioLegend, H4A3, RRID:AB_1227506) added at co-culture onset. Stained cells were acquired on a BD FACSCanto II and analyzed with FlowJo.

### HLA restriction of 91AE T cell clone

In order to determine the HLA restriction of the 91AE T cell clone, we first performed co-cultures of the clone with the TAP1^KO^ SW480 cell line pretreated with IFN-γ, as described previously, with or without any of the following blocking mAbs (10 μg/mL final): anti-HLA-I (local production, clone W6/32), anti-HLA-A2 (BioLegend, clone BB7.2, RRID: AB_1659243), anti-HLA-A24 (local production, clone AH-195), and anti-HLA-BC (local production, clone B1.23.2). Once HLA-BC restriction had been determined, we then evaluated the clone’s recognition of the HLA-B∗0702^KO^ or HLA-Cw∗0702^KO^ TAP1^KO^ SW480 cell lines that we had generated, pretreated with IFN-γ.

### Tumor spheroid generation and analysis

#### Tumor spheroid generation

To generate spheroids from CRC lines, 200 SW480 cells (WT or TAP1^KO^) were seeded per well in a 96 ultra-low attachment multi-well plate (Corning) in 150 μL of medium with 30 μg of collagen-I per 10,000 cells (Gibco, A10483-01) at D0. On day 3, the cells were treated or not with 500 U/mL of recombinant human IFN-γ (Eurobio).

#### T cell infiltration and tumor cell apoptosis in spheroid models

Five days after spheroid initiation, a fluorogenic peptide caspase 3/7 substrate, Nucview 488 (Biotum, 10402), designed to detect caspase 3/7 activity, was added to the spheroid culture medium at a final concentration of 5 μM. The 91AE clone was also added at different E:T ratios (E:T 1:1, 2:1, and 5:1), after being labeled with Cytolight cell tracker (Sartorius, Incucyte Cytolight Rapid Red Dye, 4706) at a concentration of 0.35 μM for 20 min at 37°C with agitation. The co-culture plate was incubated in the Cell Discoverer 7 microscope (CD7, Zeiss) for 48 h, during which images were recorded every hour and compiled in Zen software to create time-lapse videos. The fluorescence intensity of Cytolight (Cy5) and NucView (GFP) was calculated using Arivis software.

### TNF-α secretion by ELISA

TNF-α release was quantified in supernatants from spheroids harvested on day 7 (i.e., 2 days after addition of the T cell clone) by ELISA (Invitrogen), according to the manufacturer’s recommendations.

### Statistics

Statistical analyses were performed using GraphPad Prism 9.2 software. All statistical details can be found in the figure legends. In all cases, a *p* value of less than 0.05 was considered statistically significant ∗, *p* < 0.01 ∗∗, *p* < 0.001 ∗∗∗, and *p* < 0.0001 ∗∗∗∗ as highly significant. ns indicates non-significant results.

## Data and code avaibility

Raw data and analyses of all experiments presented in the article are available upon reasonable request.

## Acknowledgments

The authors would like to thank Pr. Thorbald van Hall (Leiden University Medical Center, Netherlands) for providing the CRISPR-Cas9 TAP1 lentivirus and Caroline Vignes, PhD (INCIT, Nantes) for her valuable assistance with 3D imaging. The authors acknowledge the Cytocell Flow Cytometry and FACS core facility (SFR Bonamy, BioCore, Inserm UMS 016, CNRS UAR 3556, Nantes, France) for its technical expertise and help, member of the Scientific Interest Group (GIS) Biogenouest and the Labex IGO program supported by the French National Research Agency (n°ANR-11-LABX-0016-01), and the biological resource center for biobanking (CHU Nantes, Hôtel Dieu, Tumorothèque, Nantes, F-44093, France). This work was supported by grants awarded by the « Ligue contre le cancer du Grand Ouest » (Comités du Morbihan et Loir-et-Cher, 2021) and the « Ligue nationale contre le cancer “Team certification EL2022.LNCC/NaL). This work was carried out as part of the “LabEx IGO” program (ANR-11-LABX-0016-01) and then the “LabEx ImmuNE” program (ANR-16-IDEX-0007).

## Author contributions

A.M., K.D., and C.D. designed methodology, performed experiments, curated data, and analyzed data; M.D. contributed to methodological development; M.M., E.L., and R.O. performed data analyses and contributed to methodological development; E.D., P.F., and C.B. curated data and provided resources; N.L. conceived and supervised the project and edited the manuscript; H.B. performed data analysis and provided validation; A.J. designed methodology, performed experiments, curated data, analyzed data, and reviewed the manuscript; N.G.-S. conceived and supervised the project, performed project administration and funding acquisition, designed the methodology, analyzed data, and wrote and edited the manuscript.

## Declaration of interests

The authors declare no competing interests.

## Supplemental information

Video S2. Time-lapse live-cell imaging (CD7) of IFN-y-treated TAP1KO SW480 tumor spheroids co-cultured with Cytolight-labeled 91AE T cell clone, showing T-cell infiltration and NucView caspase-3-positive apoptotic tumor cells over 48h

Document S2. Article plus supplemental information
