# Supplementary material for: TAP1 deficiency reshapes tumor antigenicity and enables CD8 T cell targeting in colorectal cancer
Source: Mol Ther Oncol. 2026 Jul 17;34(3):201294. doi: 10.1016/j.omton.2026.201294 (PMC13453566; doi:10.1016/j.omton.2026.201294)
Supplement: Supplementary file 1 [file mmc1.pdf]

OMTON, Volume *34*

Supplemental information

TAP1 deficiency reshapes tumor antigenicity and enables CD8 T cell targeting in colorectal cancer

Audrey Merienne, Kathleen Ducoin, Cécile Deleine, Mathilde Deiber, Margot Machu, Edouard Leveque, Romain Oger, Emilie Duchalais, Pierre Fourquier, Céline Bossard, Nathalie Labarrière, Houssem Benlalam, Anne Jarry, and Nadine Gervois-Segain

Supplemental Materials and Methods

Generation and validation of HLA-B*07:02^{KO} and HLA-Cw07:02^{KO} SW480 cell lines

The HLA-B*07:02^{KO} or HLA-Cw07:02^{KO} TAP1^{KO} SW480 cell lines were obtained after electroporation (BTX ECM830) of ribonucleic complexes with HLA-B sgRNA (IDT, Hs.Cas9.HLA-B.1.AB, final concentration 0.45µM) or HLA-C sgRNA (IDT, Hs.Cas9.HLA-C.1.AA, final concentration 0.45µM) and Cas9 protein (IDT, S.p. HiFi Cas9 Nuclease V3, final concentration 0.3µM) in 100 µL of Optimem (Gibco). Efficacy of HLA-B*07:02 or HLA-Cw07:02 editing was assessed by flow cytometry respectively with anti-human-HLA-B7-PE mAb (Biolegend, clone BB7.1, RRID: AB_2650773) or after two-step staining with unconjugated anti-human HLA-BC primary mAb (local production, mouse, clone B1.23.2), followed with F(ab')2-Goat anti-Mouse-PE (eBioscience, RRID: AB_11044909). In both cases, staining was performed for 30 minutes at 4°C, followed by washes in PBS 0.1% BSA. Cells were analyzed on a BD FACSCanto II flow cytometer using FlowJo software.

Supplemental tables

Supplemental Table S1: Semi-quantitative immunohistochemistry of TAP1, HLA-Ia and β2m expression by tumor cells in CRC.

**Percentage of positive tumor or normal epithelial cells (score)**	**Staining intensity (score)**	**Final score**
**0**: no	**0**: no	**Score %** **X** **Score intensity**
**1**: 1 to 25%	**1**: low to medium	
**2**: 26 to 75%		
**3**: >75%	**2**: strong	

Supplemental Table S2: Correlation between the frequency of CD8 TILs reactive to WT SW480 and *in situ* TAP1 expression score in the original tumor.

	**Reactivity of CD8 TIL populations**	**TAP1 score (*in situ*) of tumors from which TILs originate**		**Chi-square test**
		**Score ≤ 2**	**Score > 2**	
**CRC (n=56)**	yes	9	31	ns p=0,601
	no	6	15	
**MSS (n=42)**	yes	7	18	ns p=0,746
	no	4	13	
**MSI (n=14)**	yes	2	8	ns p=0,261
	no	2	2	

Supplemental figures

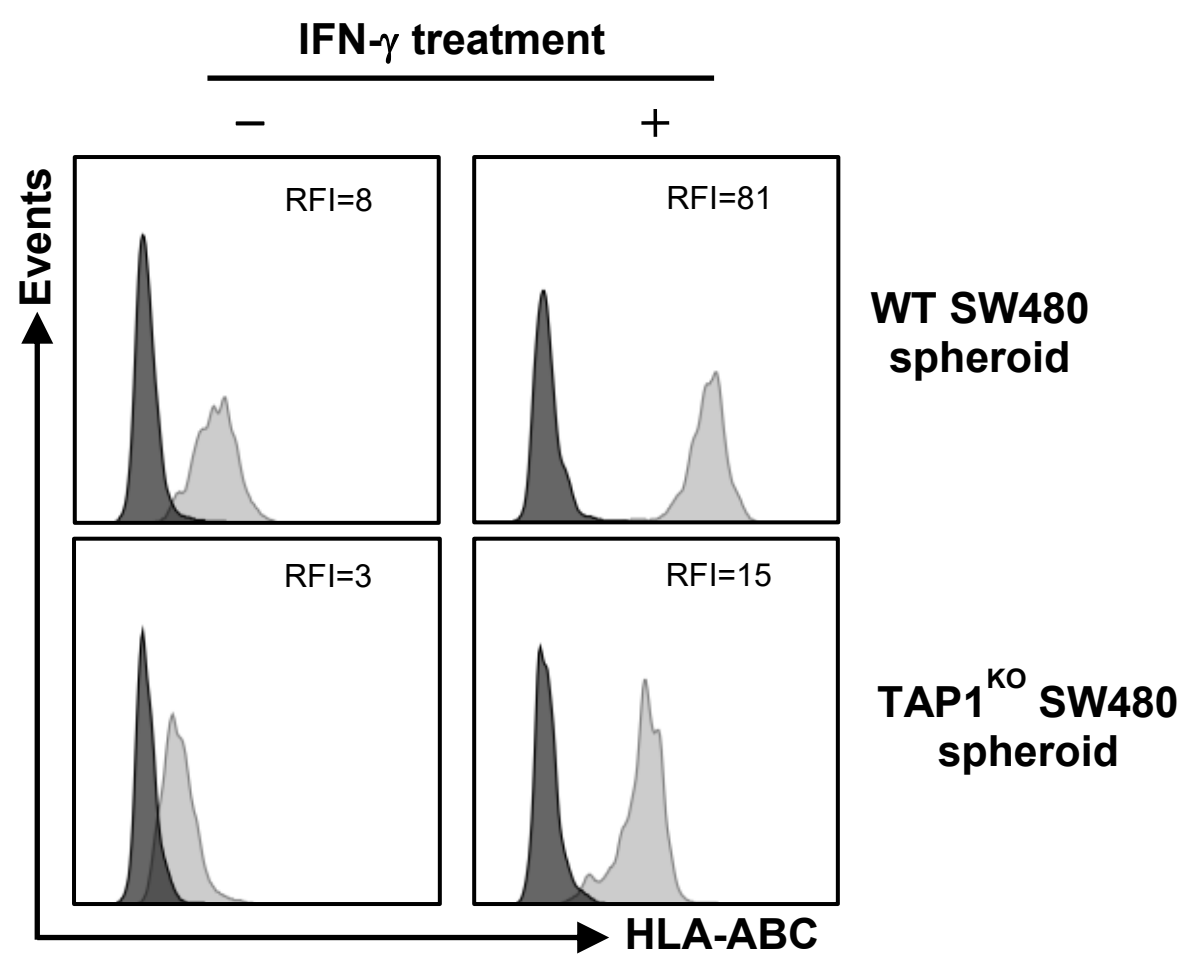

Supplemental Figure S1: HLA-ABC expression in SW480 WT or $TAP1^{KO}$ spheroids. Representative histogram illustrating HLA-ABC expression (gray) by tumor cells obtained after the dissociation at day 5 of WT or $TAP1^{KO}$ SW480 spheroids, with controls overlaid in black. Results are expressed as relative fluorescence intensity (RFI) measured by flow cytometry.

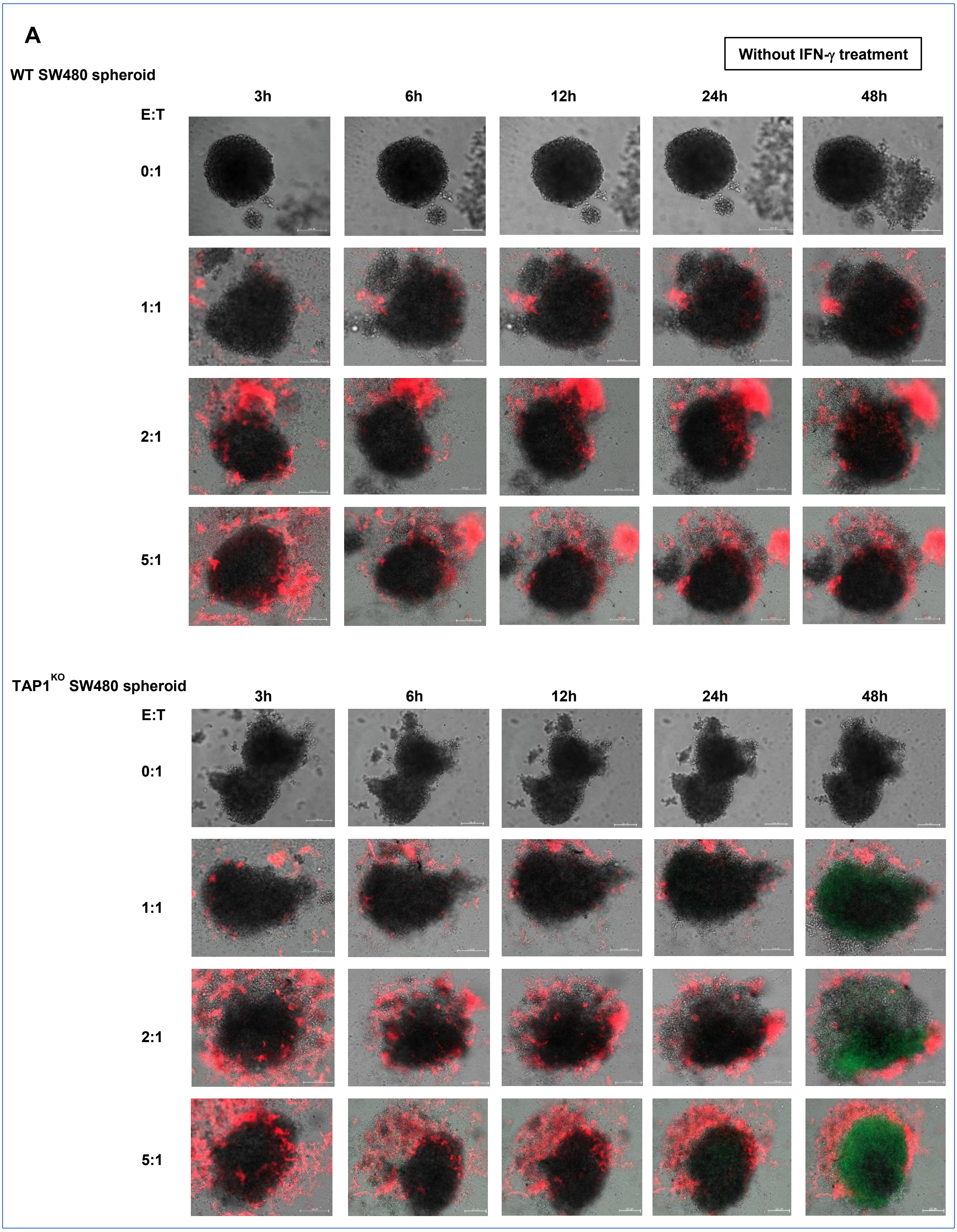
A
Without IFN-γ treatment
WT SW480 spheroid
3h
6h
12h
24h
48h
E:T
0:1
1:1
2:1
5:1
TAP1KO SW480 spheroid
3h
6h
12h
24h
48h
E:T
0:1
1:1
2:1
5:1

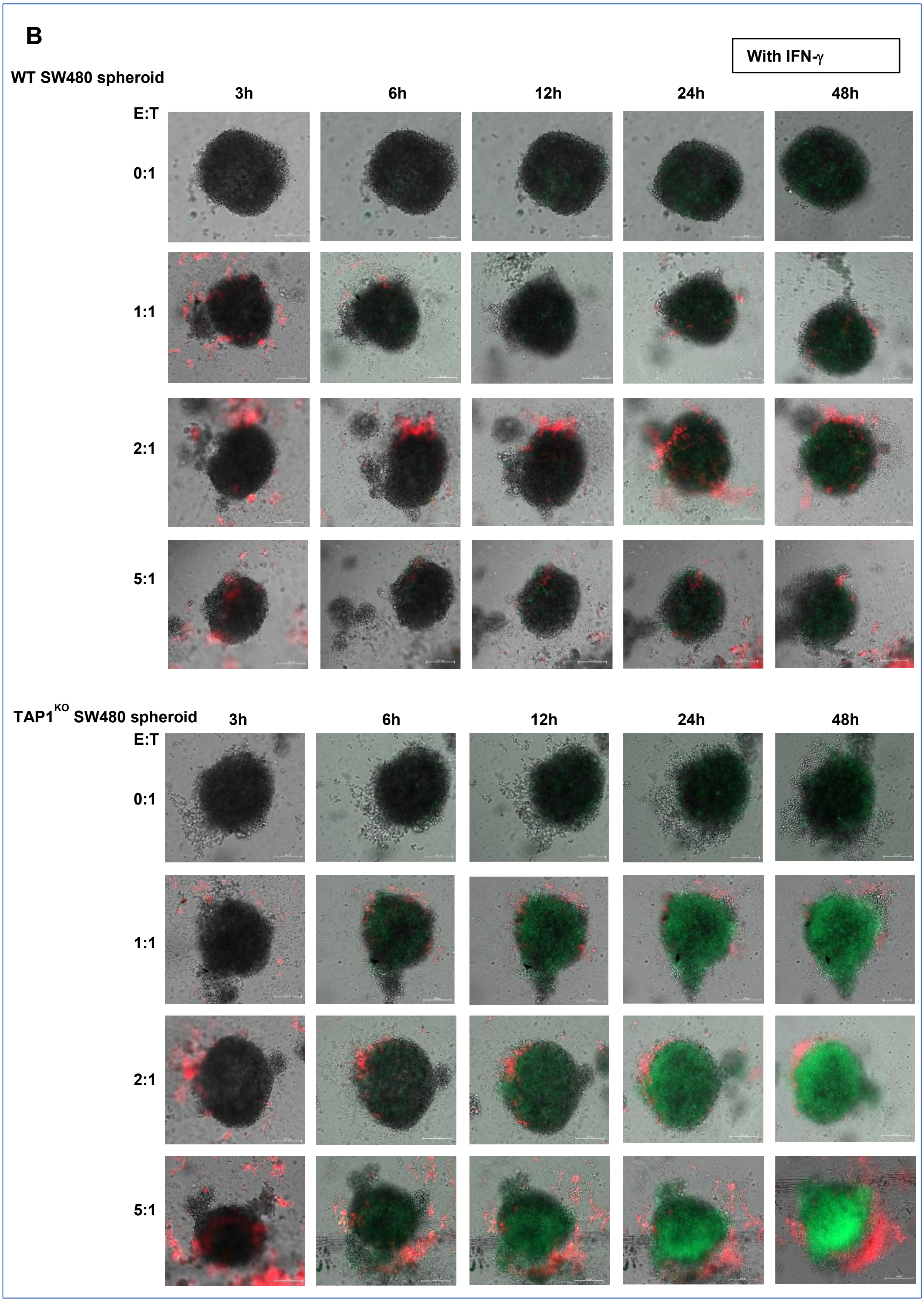
B
With IFN-γ
WT SW480 spheroid
E:T
3h
6h
12h
24h
48h
0:1
1:1
2:1
5:1
TAP1KO SW480 spheroid
E:T
3h
6h
12h
24h
48h
0:1
1:1
2:1
5:1

Supplemental Figure S2: Antitumor activity over time of a 91AE T-cell clone in a 3D spheroid model of TAP1-deficient CRC cells. Spheroid images were acquired using the Celldiscoverer 7 automated high-resolution microscope. Representative images of WT or $TAP1^{KO}$ SW480 spheroids, either without (A) or with (B) IFN-γ treatment, after 3, 6, 12, 24, and 48h of co-culture with the 91AE T-cell clone at different E:T ratios (0:1, 1:1, 2:1, 5:1). Lymphocytes are labeled in red (Cytolight) and apoptotic tumor cells in green (Nucview).

Supplemental videos

Videos S1 and S2: CD8 T cell-mediated killing of SW480 tumor spheroids visualized by live-cell caspase-3 imaging. Time-lapse live-cell imaging (CD7) of IFN-γ-treated WT or $TAP1^{KO}$ SW480 tumor spheroids (videos 1 and 2, respectively) co-cultured with Cytolight-labeled 91AE T-cell clone, showing T-cell infiltration and NucView caspase-3–positive apoptotic tumor cells over 48h.
